# Dialyzable Leukocyte Extract (Transferon™) Administration in Sepsis: Experience from a Single Referral Pediatric Intensive Care Unit

**DOI:** 10.1155/2019/8980506

**Published:** 2019-06-23

**Authors:** Maria Isabel Castrejón Vázquez, Aldo Arturo Reséndiz-Albor, Mario A. Ynga-Durand, Ivonne Maciel Arciniega Martínez, Vanessa Ivonne Orellana-Villazon, Carlos Alberto García López, Maria Laura Laue Noguera, Maria Eugenia Vargas Camaño

**Affiliations:** ^1^Department of Clinical Immunology and Allergy, Centro Médico Nacional “20 de Noviembre,” Instituto de Seguridad y Servicios Sociales de los Trabajadores del Estado, Mexico City, Mexico; ^2^Laboratorio de Inmunidad de Mucosas, Sección de Investigación y Posgrado, Escuela Superior de Medicina, Instituto Politécnico Nacional, Mexico City, Mexico; ^3^Sección de Posgrado e Investigación, Escuela Superior de Medicina, Instituto Politécnico Nacional, Mexico City, Mexico; ^4^Department of Pediatric Intensive Care Medicine, Centro Médico Nacional “20 de Noviembre,” Instituto de Seguridad y Servicios Sociales de los Trabajadores del Estado, Mexico City, Mexico

## Abstract

Immunomodulatory agents have been proposed as therapeutic candidates to improve outcomes in sepsis. Transferon™, a dialyzable leukocyte extract (DLE), has been supported in Mexico as an immunomodulatory adjuvant in anti-infectious therapy. Here we present a retrospective study describing the experience of a referral pediatric intensive care unit (PICU) with Transferon™ in sepsis. We studied clinical and laboratory data from 123 patients with sepsis (15 in the DLE group and 108 in the control group) that were admitted to PICU during the period between January 2010 and December 2016. Transferon™ DLE use was associated with lower C reactive protein (CRP), increase in total lymphocyte counts (TLC), and decrease in total neutrophil count (TNC) 72 hours after Transferon™ DLE administration. The control group did not present any significant difference in CRP values and had lower TLC after 72 hours of admission. There was no difference in PICU length of stay between control and Transferon™ DLE group. Transferon™ DLE administration was associated with a higher survival rate at the end of PICU stay. This study shows a possible immunomodulatory effect of Transferon™ on pediatric sepsis patients.

## 1. Introduction

Sepsis has been defined as a systemic inflammatory response syndrome in the presence of a suspected or proven infection [1]. It is a frequent cause of admission in pediatric inpatient care units and the leading cause of death among hospitalized children across different settings [2, 3]. Recent guidelines have established initial management recommendations which emphasize early recognition, fluid resuscitation, antibiotic administration, and inotropic infusion if needed [4]. Definitive care usually takes place in a pediatric intensive care unit (PICU) where advanced interventions such as cardiopulmonary mechanical support are initiated. Sepsis-associated cardiovascular dysfunction, also referred to as septic shock, has a higher mortality and worse long-term outcomes than sepsis alone [5]. PICU-derived interventions could result in complications such as ventilator-associated pneumonia and catheter-associated infections that contribute further to worse outcomes. Immunosuppression has been suggested as an important factor in intensive care unit-acquired infections and less overall survival [6]. Despite improvements in medical care, new therapeutic approaches are needed.

It has suggested that sepsis may be at its core an immune dysregulation entity [7]. The degree of early hyperinflammation after initial infectious insult is associated with worse adverse outcomes [8]. Nevertheless, mounting evidence suggests that a compensatory anti-inflammatory response develops at the same time, and its persistence and severity represents a form of acquired immunodeficiency that has been termed immunoparalysis [9, 10]. The innate and adaptive arms of the immune system are affected by this phenomenon [11]. Lymphocyte suppression has been repeatedly associated with adverse outcomes and it has been suggested that a reversal of immunoparalysis could be obtained by immunomodulatory treatment such as granulocyte macrophage colony-stimulating factor (GM-CSF) [12], anti-PD-L1 monoclonal antibody [13], and recombinant human IL-7 [14]. Immunostimulation in the presence of immunoparalysis is a promising new venue for research [15].

Dialyzable leukocyte extracts (DLE) are heterogeneous mixtures of low-molecular-weight peptides (<10 kDa) that are released on disruption of peripheral blood leukocytes from healthy subjects [16]. Several patented processes for DLE production are available, including a human derived DLE [17]. Administration of DLE has been reported as an effective adjuvant in the treatment of infections, allergies, cancer, and immunodeficiencies [18]. DLE ability to modulate immune responses has been defined in several reviews as due to immune activator and suppressor properties. The activator portion sets the immune system in a state of readiness [19]. When nonimmune leukocyte populations are under the influence of DLE, they acquire an improved capacity to respond to specific antigens. It enhances the antigenic stimulus which boosts the production of interferon gamma (IFN-*γ*), interleukin (IL)-2, IL-17, and tumor necrosis factor alpha (TNF-*α*) by CD4+ T cells [20, 21]. Consequently, improved cell-mediated immune response develops against the target antigen. DLE effects on Toll-like receptors (TLR2 and TLR4) and nuclear factor kappa-light-chain-enhancer of activated B cells (NF-*κ*B) expression and its regulation on TNF-*α*, IL-6, and IL-8 production have been described [22, 23]. The suppressor component maintains a balance in the immune system, preventing its overactivity in the absence of any new threats. DLE suppressor components are involved in the regulation of the immune response to antigen by modulating the production of IL-10 [24]. Hematopoietic activity can also be detected in DLE as well as bactericidal and bacteriostatic properties [25–28].

DLE use as an immunomodulator has been recommended as adjuvant treatment in several infectious and immunological diseases and other conditions such as sepsis, major surgery recovery, and various neoplasias. These clinical guidelines from Mexico were developed based on local expert's opinion and experience with Transferon™ [29]. Transferon™, a DLE manufactured in Mexico, has been used from more than two decades in oral and parenteral presentations. It is produced under Good Manufactoring Practice processes and facilities. Serious adverse events had not been reported and a recent safety assessment has confirmed that adverse events associated with Transferon™ are rare and nonserious [30]. Transferon™ has been approved by the Mexican drug regulatory agency (COFEPRIS) for human consumption as an immunomodulatory agent, and a robust biological assay has been developed to serve as a quality control, ensuring an adequate and measurable activity of Transferon™ [31]. In preclinical studies, Transferon™ has shown to induce activation of TLR-2 signaling in monocytes [32] and, recently, its effect on the expression of costimulatory molecules CD80 and CD86 and the secretion of IL-6 in lipopolysaccharide- (LPS-) activated macrophages [33].

DLEs are not included in the international guidelines on sepsis management and it has been ignored as a treatment option. Despite locally generated clinical recommendations and apparent safe profile, adequate clinical studies are lacking regarding DLE efficacy, safety, and mechanism of action in sepsis. The main aim of this research is to describe the experience of a referral pediatric PICU with this immunomodulatory agent by conducting a retrospective study in order to ascertain the immunological and clinical effect of DLE.

## 2. Population and Methods

### 2.1. Study Location

Centro Medico Nacional 20 de Noviembre's Pediatric Intensive Care Unit (CMN20NOV-PICU) is a third level referral center located in Mexico City. Its patient population is characterized by complex multimorbidity with most of the cases being admitted due to complications related to oncohematology treatment or critical surgery recovery. This PICU is unique among other centers from Mexico as Transferon™ has been selectively used as an adjuvant treatment of sepsis through early consultation with the Clinical Immunology Department.

### 2.2. Study Design

This was a retrospective study combining hospitalization data from CMN20NOV-PICU clinical database and CMN20NOV electronic chart archives. The CMN20NOV-PICU is a department database containing basic information about all PICU admissions (patient data, diagnosis at PICU admission, dates of hospitalization, and vital status at discharge). Electronic charts archives contained laboratory values, comorbidities, and medications were used. The period January 2010–December 2016 was chosen because this was the period during which Transferon™ was available for treatment. This retrospective study was approved by the CMN20NOV Institutional Review Board and Ethics Committee (Registration Number 223.2015).

### 2.3. Patient Selection and Clinical Characteristics

A primary search was performed selecting patients with diagnosis of sepsis and septic shock excluding those patients with primary immunodeficiencies.

Attending physicians followed diagnostic criteria according to international guidelines [34].

- Sepsis: systemic inflammatory response syndrome (SIRS) in the presence of or as a result of suspected or proven infection.
- Infection: a suspected or proven (by positive culture, tissue stain, or polymerase chain reaction test) infection caused by any pathogen OR a clinical syndrome associated with a high probability of infection. Evidence of infection includes positive findings on clinical exam, imaging, or laboratory tests (e.g., white blood cells in a normally sterile body fluid, perforated viscus, chest radiograph consistent with pneumonia, petechial or purpuric rash, or purpura fulminans)
- SIRS: the presence of at least two of the following four criteria, one of which must be abnormal temperature or leukocyte count:
  - Core temperature of >38.5°C or <36°C.
  - Tachycardia, defined as a mean heart rate >2 SD above normal for age in the absence of external stimulus, chronic drugs, or painful stimuli, or otherwise unexplained persistent elevation over a 0.5-to-4-hr time period OR for children <1 yr old: bradycardia, defined as a mean heart rate <10th percentile for age in the absence of external vagal stimulus, *β*-blocker drugs, or congenital heart disease, or otherwise unexplained persistent depression over a 0.5-hr time period.
  - Mean respiratory rate >2 SD above normal for age or mechanical ventilation for an acute process not related to underlying neuromuscular disease or the receipt of general anesthesia.
  - Leukocyte count elevated or depressed for age (not secondary to chemotherapy-induced leukopenia) or >10% immature neutrophils.
- Septic shock: sepsis and cardiovascular organ dysfunction as defined as the presence of the following criteria despite administration of isotonic intravenous fluid bolus ≥40 mL/kg in 1 hr:
  - Decrease in BP (hypotension) 5th percentile for age or systolic BP 2 SD below normal for age
- OR
  - Need for vasoactive drug to maintain BP in normal range (dopamine 5 g/kg/min or dobutamine, epinephrine, or norepinephrine at any dose)
- OR
  - Two of the following:
    1. Unexplained metabolic acidosis: base deficit >5.0 mEq/L.
    2. Increased arterial lactate >2 times upper limit of normal.
    3. Oliguria: urine output 0.5 mL/kg/hr.
    4. Prolonged capillary refill: >5 secs.
    5. Core to peripheral temperature gap >3°C.

Primary immunodeficiency was defined by the presence of a previously established diagnosis by a clinical immunology specialist.

Admitted patients were divided into two groups based on Transferon™ DLE use. DLE group were defined as those receiving Transferon™ at any time during their PICU stay. Usual administration of such intervention is done in the first 72 hours according to institutional guidelines through early consultation with Clinical Immunology Department. Information of the admission day, day 3 after admission or DLE administration (depending on the group), and final day of hospitalization were collected, as well as clinical and laboratory data. Outcomes of interest were PICU mortality, PICU length of stay, PICU days under ventilatory support and PICU days under pharmacologic cardiovascular support (inotropes and vasoactive drugs).

### 2.4. Serum Biomarkers

Laboratory data of interest were C reactive protein (CRP), erythrocyte sedimentation rate (ESR), total lymphocyte count (TLC), and total neutrophil count (TNC). These biological measurements were done as part of clinical monitoring in a pediatric critical care unit. As a routine laboratory practice in the institution where this study took place, an automatized system was used to measure the serum CRP by means of a turbidimetric method. ESR was measured by Westergren method [35]. TLC and TNC were measured by automatized system based on electrical impedance. Patients whose files were not available due to administrative or legal reasons, as well as those without minimal laboratory data, were not included

### 2.5. Dialyzable Leukocyte Extract (Transferon™)

Transferon™ is a DLE manufactured by the National School of Biological Sciences (ENCB) of the National Polytechnic Institute (IPN) in Mexico, at facilities that comply with good manufacturing practices by international guidelines. The active pharmaceutical ingredient of Transferon™ is based on peptides polydispersion that have been extracted from lysed human leukocytes by a dialysis process and a subsequent ultrafiltration step to select molecules below 10 kDa [36]. Further physicochemical characterization showed batch-to-batch consistency in peptide hydrophobicity, chemical composition, and molecular mass. Transferon™ is registered by Mexican health authorities as a medical drug and commercialized nationwide [37]. Transferon™ has been selectively used as an adjuvant treatment of sepsis through early consultation with the Clinical Immunology Department at CMN20NOV-PICU following locally generated guidelines mentioned above.

### 2.6. Statistical Analysis

Data distribution was analyzed using Shapiro-Wilk normality test with Royston method to determine if parametric or nonparametric evaluation should be used. All datasets followed a nonparametric distribution. Nonparametric paired datasets were compared using Wilcoxon matched pairs test. Every time a statistically significant difference was found, the median of differences is reported. Unpaired datasets were compared using Mann-Whitney test. A value of P < 0.05 was considered statistically significant. In case of categorical data (sex, presence of hemato-oncologic comorbidity, presence of septic shock, and vital status at the end of PICU stay), a chi-square test with Yates continuity correction was performed. Odd Ratio (OR) for vital status at the end of PICU stay was analyzed by Baptista-Pike method. Statistical analysis was performed by using GraphPad Prism 6 software package (GraphPad Software Inc, San Diego, CA, USA).

## 3. Results

Of the 133 patients with sepsis and septic shock included in the CMN20NOV-PICU clinical database, 123 patients met the entry criteria for the present study. Of these 15 (12.2%) received Transferon™ DLE (Table 1). When comparing the control group, patients in the Transferon™ DLE group had higher TNC at PICU admission. There were not significantly different in age, hemato-oncologic comorbidity, presence of septic shock, or ESR, CRP, and TLC values at baseline.

**Figure 1:**
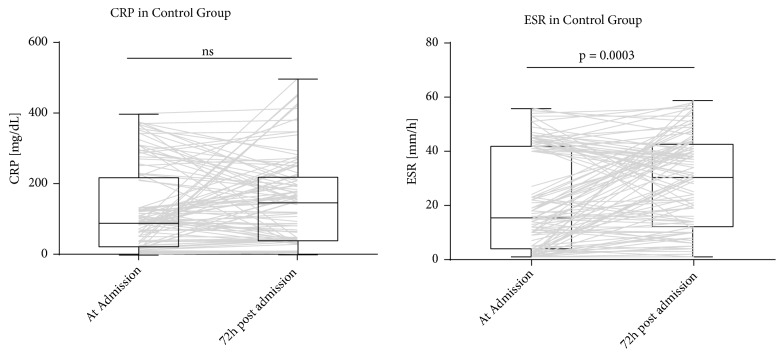
CRP and ESR in control group at admission and 72 hours after admission. Gray lines show individual trajectories.

In the control group, CRP and ESR levels were compared at admission and at 72 hours after admission (Figure 1). There was no significant difference in CRP values (95%CI -1 to 16, p=0.1259). ESR at 72 hours was significantly different from admission values (median of differences 2, 95%CI 0 to 6, p=0.0003).

**Figure 2:**
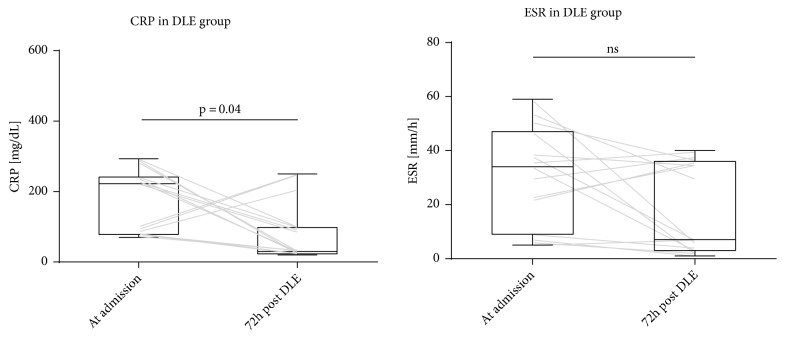
CRP and ESR in DLE group at admission and 72 hours after DLE administration. Gray lines show individual trajectories.

In the DLE group, we compared CRP and ESR levels at admission and 72 hours after DLE administration (Figure 2). A lower CRP at 72 hours after DLE administration compared to CRP at admission was found (median of differences -138, 95%CI -201 to -40, p=0.0413). There was no significant difference in ESR values (95%CI -31 to 4, p=0.075).

**Figure 3:**
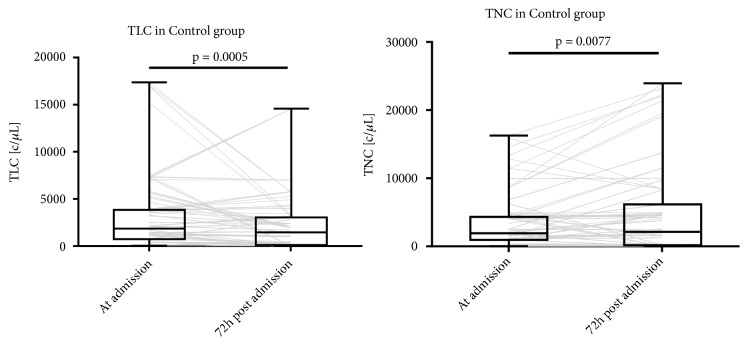
TLC and TNC in control group at admission and 72 hours after admission. Gray lines show individual trajectories.

In the Control group, we compared TLC and TNC levels at admission and at 72 hours after admission (Figure 3). TLC at 72 hours was significantly lower than admission values (median of differences -210, 95%CI -331 to -160, p=0.0005). TNC at 72 hours was significantly different from admission values (median of differences 75, 95%CI -40.00 to 376, p= 0.0077).

**Figure 4:**
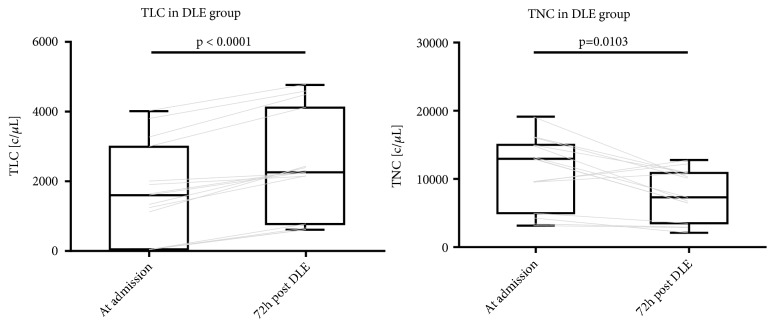
TLC and TNC in DLE group at admission and 72 hours after admission. Gray lines show individual trajectories.

In the DLE group, we compared TLC and TNC levels at admission and at 72 hours after DLE administration (Figure 4). TLC at 72 hours was significantly higher than admission values (median of differences 734, 95%CI 578.0 to 1062, p < 0.0001). TNC at 72 hours was significantly lower than admission values (median of differences -4076, 95%CI -6468 to -277.0, p= 0.0103).

**Figure 5:**
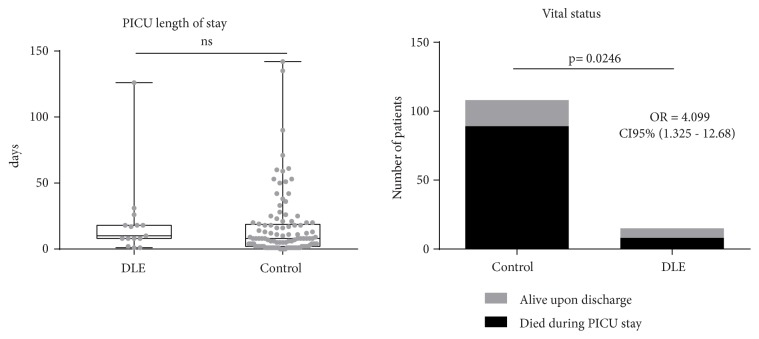
PICU length of stay and vital status at the end of PICU stay in DLE and control group.

There was no difference in days under vasoactive drugs (p=0.2625 CI 95% -7 to 1). A shorter use of ventilatory support was found in control group (median of difference -7.5, p=0.0341 CI 95% -9 to 0). There was no difference in PICU length of stay between control and DLE group (p=0.2779 CI95% -8 to 2). Of the 108 subjects from the control group, 89 (82.41%) died during PICU stay. In DLE group 8 out of 15 subjects died during PICU stay (53.33%). DLE administration was associated with a higher survival rate (OR 4.099 CI95% 1.325 to 12.68, p=0.0246) (Figure 5).

## 4. Discussion

DLE therapeutic effect on immune dysregulation states such as sepsis and septic shock has been proposed based mostly on its anti-inflammatory properties. DLE has shown to reduce TNF-*α* and IL-6 in human whole blood cells after stimulation with LPS. Surprisingly, IL-10, an anti- inflammatory cytokine, was also reduced [24]. DLE inhibitory effect on TNF-*α*'s whole blood production after LPS stimulation was confirmed by Ojeda et al., but cell-specific action is diverse. They found that DLE diminished TNF-*α* production in LPS-stimulated monocytes and leukocytes, while TNF-*α* was increased in endothelial cells [23]. DLE effect on cytokines could also be related to the specific activation state of cells, DLE increased TNF-*α* and IL-6 in resting human macrophages while after LPS stimulation DLE treatment reduced TNF-*α* and IL-6 production. In both resting and LPS-stimulated macrophages, DLE increased IL-10 production [38]. NF-*κ*B inhibition has been proposed as the mechanism of this immunomodulatory effect [39]. In a LPS-induced murine shock model, DLE administration suppressed TNF-*α*, IL-6, and IL-10 mRNA expression in the spleen as well as reducing blood levels. DLE reduced mortality from 100% to 20% in this particular endotoxemia model [40]. These data suggest that DLE could act as an anti- inflammatory or proinflammatory immunomodulator in immune-evolving pathologies such as sepsis. Isolated clinical reports support DLE administration in sepsis and one study in neonatal sepsis showed an increase in leukocyte numbers and survival [41, 42]. It is important to emphasize that these studies differ on the origin of the leukocytes and subsequent processing of the DLE and may not be completely comparable. It is possible that the biological material or manufacturing steps could have an impact on the mechanism of action or target specificity, but this question has not been addressed in a definitive way. In this regard, Jimenez-Uribe and collaborators found that simultaneous stimulation of macrophage-like cells derived from THP-1 monocytes with Transferon™ and LPS elicited a significant increase in CD80 and CD86 expression, as well as in IL-6 production compared to the LPS control [33] in contrast to previously discussed results from bovine-originated DLE [24]. This finding stresses the importance of specific-DLE research to delineate differences between products. It is possible that the interpretation of our findings will be only attributable to Transferon™.

In our study, we collected data from pediatric patients admitted to a third level referral central. We divided them in two groups based on Transferon™ administration and compared inflammatory biomarkers and clinical outcomes. Control group's CRP values did not show difference between admission levels and levels measured 72 hours later, while Transferon™ DLE group was associated with lower CRP measured 72 hours after treatment. Since CRP is an acute phase reactant produced in the liver by IL -6 stimulation, it could be used as a surrogate marker of unspecific inflammation [43]. CRP is particularly helpful in the evaluation of immunosuppressed individuals, not being affected by medications or hematologic conditions [44, 45]. Increased CRP levels in sepsis are associated with worse prognosis [46]. Transferon™ could have exerted immunomodulatory effects on septic patients, changing the evolution of the inflammatory response as showed by other DLEs in murine sepsis models.

Neutrophil role as a biomarker in sepsis is complex [47]. They are crucial components of the innate immune response during sepsis, releasing important regulatory cytokines, chemokines, and leukotrienes, contributing directly to antimicrobial killing and resolution of infections. Nonsurviving sepsis patients showed lower neutrophil counts in blood than survivors at clinical diagnosis in an adult population [48]. This could be related to secondary immunosuppressive conditions, redistribution of neutrophils from blood to tissues and endothelia, and insufficient bone marrow production. On the other hand, increased presence of immature forms of neutrophils in the blood of septic patients has been associated with severe disease. It has been showed that a significant increase in the neutrophil count was present in nonsurvivors compared with survivors [49]. As a consequence, TNC measurements are variable and without a clear prognosis significance. In this study, TNC from control group was significantly different after 72 hours from admission, but 95% confidence interval for the median of the paired differences ranged from positive to negative effect. Transferon™ DLE group TNC showed a significant difference after treatment, showing a diminishing trend. The combination of lower inflammation biomarkers such as CRP and lower neutrophil counts may be a better representation of improvement against infectious complications [50], and we found this combination in the intervention group.

An important feature of sepsis-induced immunosuppression is apoptosis-related loss of immune cells. Clinical studies have previously demonstrated that circulating levels of lymphocytes fall during the onset of sepsis and can remain depressed for up to 28 days despite standard treatment [51]. Prevention of lymphocyte cell death in murine sepsis models had shown a positive impact on survival [52]. Persistent lymphopenia on the fourth day following the diagnosis of sepsis predicts early and late mortality in adult patients [53]. Prolonged lymphopenia is a candidate marker of persistent immunosuppression in septic patients, and absolute lymphocyte counts are easily measured during routine care. DLE therapy has previously shown a positive effect on total lymphocyte counts in HIV infected individuals [54] and cancer patients [55]. In our study, TLC from control group was significantly lower after 72 hours compared to admission, while Transferon™ therapy was associated with an improvement in absolute numbers. This data, added to changes in CRP, supports the possible Transferon™ effect on lymphocytes recovery.

Finally, we looked at the effect on PICU length stay and we did not find difference in the number of days between control and Transferon™ DLE groups. It is important to distinguish that a shorter PICU stay could also be caused by an earlier death. Thereby, we looked at survival rates and we found that Transferon™ therapy was associated with a higher survival rate with an OR of 4.099. Perez and collaborators reported partial results from a small-randomized trial in 24 adult patients comparing Transferon™ vs. placebo in severe sepsis [56]. They showed that Transferon™ increased DR expression in the CD14^+^ cells, increased number of Th (CD3^+^/CD4^+^) population, decreased circulatory neutrophils percentages, and decreased the time of stay in the intensive care unit. No differences in survival were reported and full data has not been published yet. Our results are concordant with their preliminary findings except for the effect on intensive care unit length stay, which could be affected by survival, early discharge and transfer to intermediate care unit, a common practice in adult intensive medicine.

Our study has several limitations. As there is no gold standard in the definition of sepsis, clinicians have attempted to diagnose it by combining physiological and laboratory abnormalities. This nonspecific criterion is particularly problematic in pediatric population as normal values are on constant change according to age and previous comorbidities. Thus, criterion proposed by expert consensus in adult-directed conferences had been adapted by a panel of pediatric critical care specialist in 2005 [34]. This approach has been subject of debate due to its low specificity and limited practical use in clinical situations, especially in low to medium income countries. In our study, involved pediatric critical care specialists were required to follow the consensus to fulfil academic and institutional recommendations, generating a standardized and predictable diagnostic pathway that is similar to multicenter trials, although the biological homogeneity of this condition is inconclusive. Selection bias is an important threat in case-control studies and in our admission data Transferon™ DLE group had higher TNC than the control group. It is possible that clinicians intentionally selected this subset of patients for immunomodulatory therapy, as high TNC is typically associated with inflammation and thus had higher chances of receiving DLE in comparison to the lower TNC group. Since isolated TNC number, low or high, is not a solid prognostic marker, it is unclear if this selection bias is associated with higher survival per se. Regarding the dose and time of intervention, according to internal referral guidelines Transferon™ was administered in the first 72 hours of admission at the recommended dosage by the national expert consensus (1-2 units every 12 hours for 10 days) [29], but exact duration of the intervention was not available and it is possible that treatment times varied significantly among patients. This could be due to the dynamic changes in therapy, usually the case in critically ill individuals. Although this limits the interpretation of the effect size due to intervention, this report provides preliminary information that underscores the necessity of prospective studies in which a cause and effect relationship could be determined. Another caveat of our study, common in observational design, is the small number of case group size in comparison to control group (1 to 7.2). On this subject, there has been an important discussion regarding the risk of overestimation of effect size and low reproducibility with such design [56]. This is particularly key in the interpretation of Transferon™ odds ratio values related to the vital status at the end of PICU stay, which may be a “winner's course” phenomenon (studies that find evidence of an effect often provide inflated estimates of the size of that effect). This inflation is worst for small studies such us ours. In relation to the control size group, if a limited number cases is available, once past a certain point, increasing the number of controls will not add any statistical power and could be considered unnecessary but not detrimental [57]. In this regard, control misclassification could be a more significant issue, but as a retrospective study, it is not possible to completely counteract this factor. Despite these shortcomings, this experience with DLE intervention in pediatric septic patients is worth reporting and constitutes to our knowledge the first study showing the association between beneficial outcomes and immunomodulatory effect of DLE on this patient subset.

## 5. Conclusion

Our report is the first retrospective study describing a referral pediatric intensive care unit's (PICU) experience with DLE in sepsis as an adjuvant treatment to international guidelines-established management. DLE use was associated with lower C reactive protein, increase in total lymphocyte counts, and decrease in total neutrophil count. DLE administration was associated with a higher survival rate. As new therapeutic venues are actively pursued in the management of sepsis and septic shock, DLE could be an interesting strategy for improving outcomes and reducing complications.

## Figures and Tables

**Table 1:** Patient baseline characteristics at PICU admission.

	Control (n=108)	DLE (n=15)	*P*
Age, months (25p-75p)	60 (12-117)	60 (34-168)	ns

Female (%)	60 (55.6)	7 (46.7)	ns

Hemato-Oncologic comorbidity (%)	62 (57.4)	8 (53.3)	ns

Septic Shock (%)	83 (76.9)	8 (53.3)	ns

C Reactive Protein (CRP) at admission [mg/dL] (median, 25p-75p)	90 (23.5-218)	222 (78-241)	ns

ESR at admission [mm/H]	15.5 (4-42)	34 (9-47)	ns

Total Lymphocyte Count at admission (TLC) [c/uL] (median, 25p-75p)	1840 (730-3820)	1609 (46-3001)	ns

Total Neutrophil Count at admission (TNC) [c/uL] (median, 25p-75p)	1900 (940-4200)	12966 (4981-15019)	*∗∗∗* *∗*

## Data Availability

The data that support the findings of this study are available from the corresponding author upon reasonable request.
